# Persistent or recurrent Barrett’s neoplasia after an endoscopic therapy session is associated with DNA content abnormality and can be detected by DNA flow cytometric analysis of paraffin-embedded tissue

**DOI:** 10.1038/s41379-021-00832-8

**Published:** 2021-06-09

**Authors:** Christopher J. Bowman, Ruth Zhang, Dana Balitzer, Dongliang Wang, Peter S. Rabinovitch, Bence P. Kővári, Aras N. Mattis, Sanjay Kakar, Gregory Y. Lauwers, Won-Tak Choi

**Affiliations:** 1 University of California at San Francisco, Department of Pathology, San Francisco, CA USA; 2 San Francisco VA Health Care System, Department of Pathology, San Francisco, CA USA; 3 SUNY Upstate Medical University, Department of Public Health and Preventive Medicine, Syracuse, NY USA; 4 University of Washington, Department of Pathology, Seattle, WA USA; 5 H. Lee Moffitt Cancer Center, Department of Pathology, Tampa, FL USA

**Keywords:** Oesophageal cancer, Oesophageal cancer

## Abstract

Endoscopic therapy is currently the standard of care for the treatment of high-grade dysplasia (HGD) or intramucosal adenocarcinoma (IMC) in patients with Barrett’s esophagus (BE). Visible lesions are treated with endoscopic mucosal resection (EMR), which is often coupled with radiofrequency ablation (RFA). However, endoscopic therapy may require multiple sessions (one session every 2-3 months) and does not always assure complete eradication of neoplasia. Furthermore, despite complete eradication, recurrences are not uncommon. This study assesses which potential risk factors can predict a poor response after endoscopic sessions. Forty-five BE patients who underwent at least one endoscopic session (EMR alone or ablation with or without preceding EMR) for the treatment of HGD/IMC, low-grade dysplasia (LGD), or indefinite for dysplasia (IND) were analyzed. DNA flow cytometry was performed on 82 formalin-fixed paraffin-embedded samples from the 45 patients, including 78 HGD/IMC, 2 LGD, and 2 IND. Eight non-dysplastic BE samples were used as controls. Three to four 60-micron thick sections were cut from each tissue block, and the area of HGD/IMC, LGD, or IND was manually dissected. Potential associations between clinicopathologic risk factors and persistent/recurrent HGD/IMC following each endoscopic session were examined using univariate and multivariate Cox models with frailty terms. Sixty (73%) of the 82 specimens showed abnormal DNA content (aneuploidy or elevated 4N fraction). These were all specimens with HGD/IMC (representing 77% of that group). Of these 60 HGD/IMC samples with abnormal DNA content, 42 (70%) were associated with subsequent development of persistent/recurrent HGD/IMC (*n* = 41) or esophageal adenocarcinoma (EAC; *n* = 1) within a mean follow-up time of 16 months (range: 1 month to 9.4 years). In contrast, only 6 (27%, all HGD/IMC) of the 22 remaining samples (all with normal DNA content) were associated with persistent/recurrent HGD/IMC. For outcome analysis per patient, 11 (24%) of the 45 patients developed persistent/recurrent HGD/IMC or EAC, despite multiple endoscopic sessions (mean: 3.6, range: 1–11). In a univariate Cox model, the presence of abnormal DNA content (hazard ratio [HR] = 3.8, *p* = 0.007), long BE segment ≥ 3 cm (HR = 3.4, *p* = 0.002), endoscopic nodularity (HR = 2.5, *p* = 0.042), and treatment with EMR alone (HR = 2.9, *p* = 0.006) were significantly associated with an increased risk for persistent/recurrent HGD/IMC or EAC. However, only abnormal DNA content (HR = 6.0, *p* = 0.003) and treatment with EMR alone (HR = 2.7, *p* = 0.047) remained as significant risk factors in a multivariate analysis. Age ≥ 60 years, gender, ethnicity, body mass index (BMI) ≥ 30 kg/m^2^, presence of hiatal hernia, and positive EMR lateral margin for neoplasia were not significant risk factors for persistent/recurrent HGD/IMC or EAC (*p* > 0.05). Three-month, 6-month, 1-year, 3-year, and 6-year adjusted probabilities of persistent/recurrent HGD/IMC or EAC in the setting of abnormal DNA content were 31%, 56%, 67%, 79%, and 83%, respectively. The corresponding probabilities in the setting of normal DNA content were 10%, 21%, 28%, 38%, and 43%, respectively. In conclusion, in BE patients with baseline HGD/IMC, both DNA content abnormality and treatment with EMR alone were significantly associated with persistent/recurrent HGD/IMC or EAC following each endoscopic session. DNA content abnormality as detected by DNA flow cytometry identifies HGD/IMC patients at highest risk for persistent/recurrent HGD/IMC or EAC, and it also serves as a diagnostic marker of HGD/IMC with an estimated sensitivity of 77%. The diagnosis of HGD/IMC in the setting of abnormal DNA content may warrant alternative treatment strategies as well as long-term follow-up with shorter surveillance intervals.

## Introduction

Barrett’s esophagus (BE) is a genetically unstable, metaplastic epithelium that can accumulate multiple somatic mutations and chromosomal alterations, and eventually progress to dysplasia or esophageal adenocarcinoma (EAC) [1–5]. Since high-grade dysplasia (HGD) and intramucosal adenocarcinoma (IMC) are associated with a higher risk of developing EAC, endoscopic therapy is currently the standard of care for the treatment of HGD or IMC [6–11]. Visible lesions are treated with endoscopic mucosal resection (EMR), which is usually followed by radiofrequency ablation (RFA) due to increased risk of metachronous lesions in the remaining BE segment. RFA is the most widely used and preferred ablation technique, but other ablation modalities (i.e., cryotherapy, argon plasma coagulation [APC], and photodynamic therapy [PDT]) are available [8, 9, 11, 12].

Although endoscopic therapy has revolutionized the treatment of HGD/IMC, it may require multiple sessions (one session every 2-3 months) that may extend over a period of more than one year [9, 13, 14]. Despite multiple sessions, ~20% of BE patients do not achieve complete eradication of neoplasia, and recurrences are not uncommon [7, 13, 15–20]. Reasons for varying outcomes are poorly understood, but there is evidence that persistent or recurrent neoplasia following endoscopic therapy may be associated with persistent or *de novo* molecular alterations, respectively [21–23]. For instance, in a longitudinal case series of 19 BE patients with HGD/IMC undergoing RFA, Zeki et al. reported that five patients with persistent HGD/IMC had the same *TP53* or *CDKN2A* mutations in specimens taken before and after RFA/EMR [21]. Recurrent disease in three other patients was characterized by *de novo* mutations. Similarly, in a prospective study of 126 BE patients, Prasad et al. demonstrated that patients with *CDKN2A* allelic loss as detected by fluorescence in situ hybridization (FISH) had a 75% reduction in response to PDT compared to those without *CDKN2A* loss [22]. However, the utility of these molecular markers (including 9p loss of heterozygosity [LOH; site of *CDKN2A*], 17p LOH [site of *TP53*], and mutations of *TP53* and *CDKN2A*) is limited, as these changes tend to occur early and frequently, even in non-dysplastic BE, and often before DNA flow cytometric markers of neoplasia or progression (aneuploidy or elevated 4N fraction) are detectable [1–5, 24, 25].

In this regard, we recently demonstrated that the presence of DNA content abnormality as detected by DNA flow cytometry using formalin-fixed paraffin-embedded (FFPE) tissue is indicative of a higher risk for subsequent detection of HGD or EAC in BE patients with baseline low-grade dysplasia (LGD) or indefinite for dysplasia (IND) [26]. Given the need to identify these higher-risk BE patients who may either require multiple endoscopic sessions (which may increase risk of adverse events) or for whom current endoscopic therapy strategies may not be curative in the long term, we sought to determine if the finding of abnormal DNA content in HGD/IMC patients can predict a poor response following each endoscopic session. This could potentially help modify treatment strategies, especially after a few endoscopic sessions with suboptimal results. We hypothesized that HGD/IMC patients with abnormal DNA content are more likely to have persistent/recurrent HGD/IMC or EAC following an endoscopic session relative to those with normal DNA content.

## Materials and methods

### Patients and data collection

Forty-five BE patients who underwent at least one endoscopic session (EMR alone or ablation with or without preceding EMR) for the treatment of HGD/IMC, LGD, or IND between 2000 and 2019 were identified through the pathology archive of the University of California at San Francisco (UCSF) Medical Center and San Francisco VA Health Care System (Table 1). The patients underwent a total of 151 endoscopic sessions, from which 82 FFPE samples (from 82 endoscopic sessions) showed HGD/IMC (*n* = 78), LGD (*n* = 2), or IND (*n* = 2) (Table 2). Sixty-two samples were obtained from EMR, while 20 samples represented biopsies taken prior to the endoscopic sessions. For the remaining 69 endoscopic sessions, pre-endoscopic session biopsies (performed during the same ablation session) or EMR specimens showed no evidence of neoplasia or no biopsy was performed (i.e., ablation without a biopsy), so they were excluded from the study. Only the histologically confirmed HGD/IMC, LGD, or IND samples with follow-up pathology results were included. Every diagnosis was confirmed by three gastrointestinal (GI) pathologists (WTC, GYL, and BPK) using published criteria [27, 28]. If there was disagreement, consensus diagnosis was made by the three pathologists. In brief, LGD is defined by a distinct lack of surface maturation with elongated, hyperchromatic nuclei limited to the basal half of the cytoplasm, whereas HGD shows more severe cytologic and/or architectural abnormalities [27, 28]. A diagnosis of IND is usually made when the histologic differentiation of reactive inflammatory changes from dysplasia cannot be made with certainty. Relevant clinical and endoscopic data were collected by reviewing electronic medical records, including demographic risk factors (age ≥ 60 years, male gender, Caucasian ethnicity, and elevated body mass index [BMI] ≥ 30 kg/m^2^), endoscopic findings (length of BE segment [short < 3 cm and long ≥ 3 cm], nodule/nodularity, and hiatal hernia), type of endoscopic session, EMR margin status (negative or positive for neoplasia, or cannot be assessed due to fragmentation), and follow-up pathology result after each endoscopic session. Recurrent neoplasia was defined as the presence of neoplasia after complete eradication as confirmed by at least two negative follow-up endoscopic biopsies. All other cases of neoplasia detected after endoscopic sessions were considered persistent neoplasia. The study was performed with approval from the UCSF Institutional Review Board for human subjects research (IRB # 15-17416).

**Table 1:** Characteristics of BE patients who underwent at least one endoscopic session for the treatment of HGD/IMC, LGD, or IND at UCSF Medical Center and San Francisco VA Health Care System between 2000 and 2019.

Patient characteristics	Entire cohort (*n* = 45 patients)
Mean age, years (range)	67 (42–89)
Male gender, *n* (%)	41 (91%)
Caucasian race, *n* (%)	44 (98%)
Mean weight, kg (range)	89.3 (40.8–142)
Mean BMI, kg/m^2^ (range)	28.8 (17.9–41)
Hiatal hernia, *n* (%)	36 (80%)
Number of endoscopic sessions, *n* (mean, range)	151 (3.4, 1–18)

**Table 2:** Characteristics of pre-endoscopic session biopsies or EMR specimens.

Specimen characteristics	Entire cohort (*n* = 82 specimens, 45 patients)
Mean Barrett’s segment length, cm (range)	3.5 (0–11)
Nodular endoscopic appearance, *n* (%)	62 (76%)
Histologic diagnosis prior to endoscopic session, *n* (%)	
IND	2 (2%)
LGD	2 (2%)
HGD/IMC	78 (95%)
Abnormal DNA content, *n* (%)	60/82 (73%)
IND	0/2 (0%)
LGD	0/2 (0%)
HGD/IMC	60/78 (77%)
Non-dysplastic BE (*n* = 8)	0 (0%)
Type of endoscopic session, *n* (%)	
EMR alone	38 (46%)
RFA alone	17 (21%)
EMR + RFA	24 (29%)
APC or PDT alone	3 (4%)
EMR margin status, *n* (%)	
Negative	36/62 (58%)
Positive lateral margin	14/62 (23%)
Cannot be assessed due to fragmentation	12/62 (19%)
Histologic diagnosis after follow-up from endoscopic session, *n* (%)	
No dysplasia	34 (41%)
IND	0 (0%)
LGD	0 (0%)
HGD/IMC	47 (57%)
EAC	1 (1%)
Persistent/recurrent HGD/IMC or EAC after endoscopic session, *n* (%)	48 (59%)
In the setting of abnormal DNA content	42/60 (70%)
In the setting of normal DNA content	6/22 (27%)
Mean follow-up time to persistent/recurrent HGD/IMC or EAC or last biopsy, months (range)	16 (1–115)

### DNA flow cytometry

**Fig. 1:**
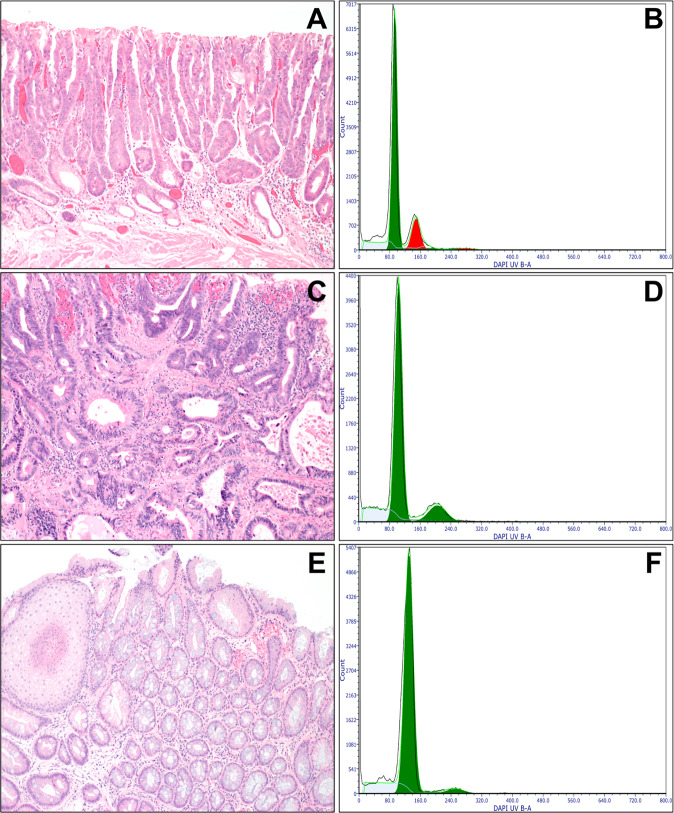
DNA content abnormality as a diagnostic marker of HGD/IMC. **A**, **B** HGD is characterized by marked cytologic and architectural atypia. The DNA histogram shows a discrete aneuploid peak (red) that is visually distinguishable from a normal diploid population (green). **C**, **D** Another example of HGD/IMC shows atypical glands lined by highly pleomorphic cells. The DNA histogram shows an elevated 4N fraction greater than 6%, but there is no distinct aneuploid peak. **E**, **F** BE without dysplasia is characterized by the presence of intestinal metaplasia. The DNA histogram shows a normal diploid population (green).

As previously described [26, 29], depending on the size of the neoplastic area, three to four 60-micron thick sections were cut from each tissue block. As the analysis of an entire biopsy or EMR specimen could dilute and mask the presence of abnormal cells present at low frequency, the area of HGD/IMC, LGD, or IND was manually dissected from the normal background mucosa to increase the sensitivity of identifying an abnormal cell population within a background of normal diploid cells. In cases where multiple biopsies obtained during the same endoscopic session or multiple tissue blocks from the same EMR specimen showed neoplasia, one tissue block with the largest neoplastic area (and the highest grade) was selected and assessed for DNA content. Eight BE samples without dysplasia were used as controls. After deparaffinization with 100% xylene and rehydration through graded ethanol to distilled water, each sample was digested with pepsin, stained with DAPI (4,6-diamidino-2-phenylindole; Accurate Chemical & Scientific Corporation, Westbury, NY), and analyzed with a BD LSRII S854 flow cytometer (BD Biosciences, San Jose, CA) using UV laser excitation. All assays were performed at UCSF. DNA content histograms were analyzed using the computer program Multicycle (De Novo software, Glendale, CA) based on the published consensus guidelines for clinical DNA flow cytometry [30]. Most epithelial cells are normally in the G_0_/G_1_ phase of the cell cycle and have diploid (2N) DNA content, while less than 6% of cells have tetraploid (4N) DNA content (G_2_). DNA aneuploidy was defined as an extra G_0_/G_1_ peak that was bimodally separated from the normal DNA diploid G_0_/G_1_ peak [30]. This is illustrated in Fig. 1B, where the approximately triploid DNA content peak is distinct and labeled in red. The finding of G_2_/tetraploid (4N) fraction greater than 6% (with DNA index of 1.9–2.1) was also classified as abnormal due to its strong association with dysplasia or EAC (Fig. 1D) [1, 26, 31, 32]. Two authors (WTC and PSR) interpreted all flow cytometric histograms independently of any other information.

### Statistical analysis

Continuous and categorical data were summarized by mean (with range) and percentage, respectively. Since many patients had two or more endoscopic sessions with different follow-up results, a frailty term with log-normal distribution was added in the univariate and multivariate Cox models to account for correlation between biopsies/EMR specimens from the same patient. Cases that did not reach the endpoint of persistent/recurrent HGD/IMC or EAC were considered as being censored in Kaplan–Meier (KM) curves and frailty models. Follow-up time was measured from the date of each endoscopic session to the date of persistent/recurrent HGD/IMC, EAC, or the last follow-up endoscopy for those in whom complete eradication of neoplasia was achieved. Adjusted probabilities of persistent/recurrent HGD/IMC or EAC at specific time points were estimated from the KM curves. Statistical significance was set at *p* < 0.05. Both 95% confidence intervals (CIs) and *p* values were calculated using the Asymptotic Wald test. Data were analyzed using SAS 9.4.

## Results

### Clinicopathologic features

Tables 1 and 2 show the clinicopathologic characteristics of our cohort who underwent at least one endoscopic session (EMR alone or ablation with or without preceding EMR) for the treatment of HGD/IMC, LGD, or IND. The patients included 41 (91%) men and 4 (9%) women with a mean age of 67 years (range: 42–89). They were predominantly Caucasian men (> 90%) with a mean BMI of 28.8 kg/m^2^ (range: 17.9–41). Hiatal hernia was common (*n* = 36; 80%). The patients underwent a total of 151 endoscopic sessions with a mean number of 3.4 sessions per patient (range: 1–18). However, only 82 FFPE samples (from 82 endoscopic sessions; mean number of samples per patient: 2, range: 1–11) showed HGD/IMC (*n* = 78; 95%), LGD (*n* = 2; 2%), or IND (*n* = 2; 2%). Sixty-two (76%) samples were obtained from EMR, while 20 (24%) samples represented biopsies taken prior to the endoscopic sessions. For the remaining 69 endoscopic sessions, there was no evidence of neoplasia on pre-endoscopic session biopsies (performed during the same ablation session) or EMR specimens, or no biopsy was performed (i.e., ablation without a biopsy). Most lesions had a nodular endoscopic appearance (*n* = 62; 76%). Of the 62 nodular lesions, EMR was performed with (*n* = 24; 29%) or without RFA (*n* = 38; 46%). Only ablation was used to treat the remaining 20 (24%) lesions, including RFA (*n* = 17; 21%), PDT (*n* = 2; 2%), and APC (*n* = 1; 1%). Thirty-six (58%) of the 62 EMR specimens showed negative resection margin(s), whereas neoplasia (LGD or HGD/IMC) was present at the lateral margin(s) in 14 (23%) EMR specimens. No deep margin was involved by neoplasia. The remaining 12 (19%) EMR specimens were too fragmented to accurately assess the margin status. The mean BE segment length prior to each endoscopic session was 3.5 cm (range: 0–11).

### DNA content analysis

**Fig. 2:**
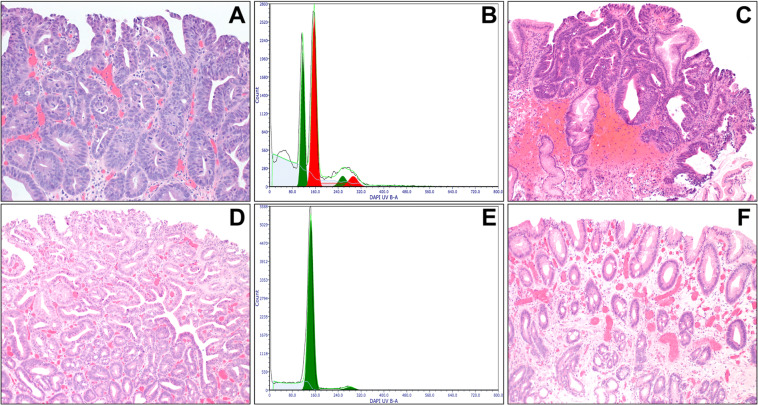
DNA content abnormality as a predictive marker of persistent/recurrent HGD/IMC or EAC. **A–C** This patient underwent EMR for HGD in November 2012 (**A**), which showed a distinct aneuploid population in the DNA histogram (red, **B**). The patient was found to have persistent HGD in January 2013 (**C**), which was removed by another EMR and two courses of RFA. The subsequent biopsies showed no evidence of neoplasia within 3 years. **D–F** Another patient underwent EMR of a nodular lesion in February 2016. The EMR demonstrated HGD (**D**) with normal DNA content in the DNA histogram (**E**). The follow-up biopsies showed no evidence of neoplasia within 4 years (**F**).

Sixty (73%) of the 82 specimens demonstrated abnormal DNA content (aneuploidy or elevated 4N fraction), and they all showed HGD/IMC (Fig. 1A–D; Table 2). Overall, 60 (77%) of the 78 HGD/IMC samples demonstrated DNA content abnormality. Of the 60 HGD/IMC samples with abnormal DNA content, 50 (83%) showed aneuploidy (Fig. 1B), whereas the remaining 10 (17%) demonstrated an elevated 4N fraction without aneuploidy (Fig. 1D). None of the 2 LGD, 2 IND, or 8 non-dysplastic BE samples showed abnormal DNA content (Fig. 1E, F). In 4 (9%) of the 45 patients, the initial biopsies/EMR specimens showed HGD/IMC with abnormal DNA content, but additional endoscopic sessions eradicated cell populations with abnormal DNA content and concomitant loss of neoplasia in subsequent biopsies (Fig. 2). Of note, 12 (86%) of the 14 EMR specimens with positive lateral margin(s), 11 (92%) of the 12 fragmented EMR specimens, and 22 (61%) of the 36 EMR specimens with negative margin(s) showed abnormal DNA content.

### Outcome analysis

After each endoscopic session, 47 (57%) of the 82 samples were associated with the detection of persistent (*n* = 44) or recurrent (*n* = 3) HGD/IMC within a mean follow-up time of 6 months (range: 1 month to 4 years). One (1%) sample was correlated with the development of EAC in 3 months (Table 2). The remaining 34 (41%) samples showed no evidence of neoplasia within a mean follow-up time of 31 months (range: 1 month to 9.4 years). Interestingly, 42 (70%) of the 60 HGD/IMC samples with abnormal DNA content were associated with the development of persistent/recurrent HGD/IMC (*n* = 41) or EAC (*n* = 1) within a mean follow-up time of 16 months (range: 1 month to 9.4 years). In contrast, only 6 (27%, all HGD/IMC) of the 22 remaining samples (all with normal flow cytometric results) were correlated with the detection of persistent/recurrent HGD/IMC. Of note, 12 (86%) of the 14 EMR specimens with positive lateral margin(s) and 7 (58%) of the 12 fragmented EMR specimens were associated with the development of persistent/recurrent HGD/IMC, whereas 18 (50%) of the 36 EMR specimens with negative margin(s) were correlated with the detection of persistent/recurrent HGD/IMC. For outcome analysis per patient, 11 (24%) of the 45 patients developed persistent/recurrent HGD/IMC or EAC, despite multiple endoscopic sessions (mean: 3.6, range: 1–11). The remaining 34 (76%) patients had no evidence of persistent/recurrent HGD/IMC, following a mean number of 3.3 endoscopic sessions (range: 1–18).

**Fig. 3:**
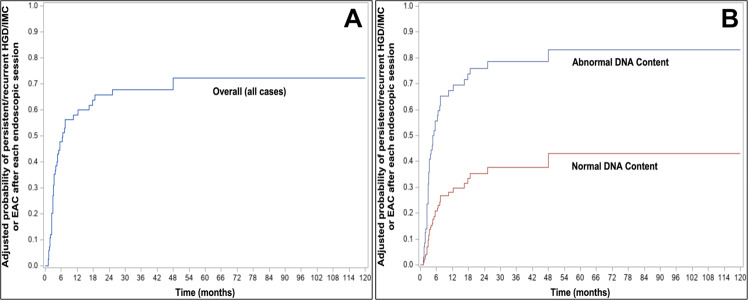
Adjusted probabilities of persistent/recurrent HGD/IMC or EAC. **A** The overall 3-month, 6-month, 1-year, 3-year, and 6-year adjusted probabilities of persistent/recurrent HGD/IMC or EAC in all patients (regardless of flow cytometric results) were 27%, 48%, 58%, 68%, and 72%, respectively. **B** Three-month, 6-month, 1-year, 3-year, and 6-year adjusted probabilities of persistent/recurrent HGD/IMC or EAC in the setting of abnormal DNA content were 31%, 56%, 67%, 79%, and 83%, respectively. In contrast, the corresponding probabilities in the setting of normal DNA content were 10%, 21%, 28%, 38%, and 43%, respectively.

The overall 3-month, 6-month, 1-year, 3-year, and 6-year adjusted probabilities of persistent/recurrent HGD/IMC or EAC in all patients (regardless of flow cytometric results) were 27%, 48%, 58%, 68%, and 72%, respectively (95% CIs = [17-36%], [36–57%], [46–67%], [56–77%], and [58–82%], respectively) (Fig. 3A; Table 3). Notably, in the setting of abnormal DNA content, the 3-month, 6-month, 1-year, 3-year, and 6-year adjusted probabilities of persistent/recurrent HGD/IMC or EAC were 31%, 56%, 67%, 79%, and 83%, respectively (95% CI = [20–41%], [42–66%], [54–77%], [64–87%], and [67–92%], respectively) (Fig. 3B; Table 3). The remaining cases with normal DNA content had corresponding probabilities of 10%, 21%, 28%, 38%, and 43%, respectively (95% CI = [1–18%], [4–35%], [6–45%], [11–57%], and [12–63%], respectively).

**Table 3:** Adjusted probabilities of persistent/recurrent HGD/IMC or EAC.

Adjusted probabilities of persistent/recurrent HGD/IMC or EAC					
Months	3	6	12	36	72
Overall (all cases) (%)	27	48	58	68	72
Abnormal DNA content (%)	31	56	67	79	83
Normal DNA content (%)	10	21	28	38	43

In a univariate Cox model, the presence of abnormal DNA content was a significant predictor of decreased response to each endoscopic session with an estimated HR of 3.8 (*p* = 0.007, 95% CI = [1.4–10.1]) (Table 4). Long BE segment ≥ 3 cm (HR = 3.4, *p* = 0.002, 95% CI = [1.6–7.6]), endoscopic nodularity (HR = 2.5, *p* = 0.042, 95% CI = [1.0–6.2]), and treatment with EMR alone (HR = 2.9, *p* = 0.006, 95% CI = [1.4–6.3]) were also significantly associated with an increased risk for persistent/recurrent HGD/IMC or EAC. However, a multivariate analysis demonstrated that only abnormal DNA content (HR = 6.0, *p* = 0.003, 95% CI = [1.8–19.6]) and treatment with EMR alone (HR = 2.7, *p* = 0.047, 95% CI = [1.0–7.0]) were significant risk factors of persistent/recurrent HGD/IMC or EAC (Table 4). Age ≥ 60 years, gender, ethnicity, BMI ≥ 30 kg/m^2^, presence of hiatal hernia, and positive EMR lateral margin for neoplasia were not significant predictors of persistent/recurrent HGD/IMC or EAC within the study period (*p* > 0.05). In another analysis after excluding all EMR specimens with positive lateral or fragmented margin(s), abnormal DNA content remained as the only significant risk factor of persistent/recurrent HGD/IMC or EAC in both univariate (HR = 3.3, *p* = 0.032, 95% CI = [1.1–9.6]) and multivariate (HR = 4.1, *p* = 0.013, 95% CI = [1.3–12.5]) Cox models (Table 5).

**Table 4:** Univariate and multivariate Cox PH models with persistent/recurrent HGD/IMC or EAC as the outcome after each endoscopic session.

	Persistent/recurrent HGD/IMC or EAC outcome		
*Univariate Cox Model*	*p* value	HR	95% CI
Abnormal flow	0.007	3.8	1.4–10.1
Normal flow			
Age ≥ 60 years	0.670	1.2	0.5–3.3
Age < 60 years			
Male	0.620	0.7	0.2–3.0
Female			
Caucasian	0.604	0.5	0.0–7.1
Non-Caucasian			
BMI ≥ 30 kg/m^2^	0.166	1.8	0.8–4.2
BMI < 30 kg/m^2^			
Long BE segment	0.002	3.4	1.6–7.6
Short BE segment			
Nodule Yes	0.042	2.5	1.0–6.2
Nodule No			
Hernia Yes	0.373	1.7	0.5–5.5
Hernia No			
EMR alone	0.006	2.9	1.4–6.3
Other modalities with or without preceding EMR			
Positive EMR lateral margin	0.081	2.1	0.9–4.8
Negative EMR margin			
*Multivariate Cox Model*			
Abnormal flow	0.003	6.0	1.8–19.6
Normal flow			
Long BE segment	0.055	2.6	1.0–6.2
Short BE segment			
Nodule Yes	0.420	1.8	0.4–7.1
Nodule No			
EMR alone	0.047	2.7	1.0–7.0
Other modalities with or without preceding EMR			
Positive EMR lateral margin	0.114	2.1	0.8–5.2
Negative EMR margin

**Table 5:** Univariate and multivariate Cox PH models with persistent/recurrent HGD/IMC or EAC as the outcome after excluding EMR specimens with positive lateral or fragmented margin(s).

	Persistent/recurrent HGD/IMC or EAC outcome		
*Univariate Cox Model*	*p* value	HR	95% CI
Abnormal Flow	0.032	3.3	1.1–9.6
Normal Flow			
Age ≥ 60 years	0.616	0.8	0.3–2.2
Age < 60 years			
Male	0.847	0.8	0.1–8.2
Female			
Caucasian	0.605	2.0	0.1–28.3
Non-Caucasian			
BMI ≥ 30 kg/m^2^	0.149	0.5	0.2–1.3
BMI < 30 kg/m^2^			
Long BE Segment	0.025	2.7	1.1–6.6
Short BE Segment			
Nodule Yes	0.136	0.4	0.2–1.3
Nodule No			
Hernia Yes	0.476	0.6	0.2–2.3
Hernia No			
EMR alone	0.024	3.0	1.2–7.6
Other modalities with or without preceding EMR			
*Multivariate Cox Model*			
Abnormal flow	0.013	4.1	1.3–12.5
Normal flow			
Long BE Segment	0.056	2.5	1.0–6.5
Short BE Segment			
Nodule Yes	0.294	0.5	0.2–1.7
Nodule No			
EMR alone	0.277	1.7	0.6–4.7
Other modalities with or without preceding EMR			

## Discussion

BE is a major risk factor for the development of EAC, especially in the presence of HGD or IMC. Although HGD/IMC was traditionally treated by esophagectomy, there is no significant difference between endoscopic therapy and esophagectomy in the rates of neoplasia remission (relative risk [RR] = 0.96) and survival (RR = ~1) [33]. Therefore, endoscopic therapy is the preferred treatment for HGD/IMC over esophagectomy [6–11]. Visible lesions require EMR, whereas flat areas can be treated with RFA. However, a significant number of BE patients (~20%) are resistant to endoscopic therapy [7, 13, 15–20] and often require multiple sessions (one session every 2-3 months) that may extend over a period of more than one year [9, 13, 14]. Even though some studies demonstrated that persistent or *de novo* molecular alterations (such as *CDKN2A* allelic loss and mutations of *TP53* or *CDKN2A*) may be responsible for persistent or recurrent neoplasia, respectively, following endoscopic therapy [21–23], the potential value of these molecular markers is limited because these changes occur early and frequently throughout large areas of BE, before the first histologic sign of dysplasia, or before the emergence of markers of neoplasia or progression as detected by DNA flow cytometry (aneuploidy or elevated 4N fraction) [1–5, 24, 25]. Identification of new biomarkers that can predict a poor response to endoscopic sessions may help identify BE patients who may benefit from alternative treatment strategies as well as long-term follow-up with shorter surveillance intervals.

In this regard, we note that DNA content abnormality in baseline HGD/IMC is a significant predictor of persistent/recurrent HGD/IMC or EAC following each endoscopic session, with an estimated HR of 6.0 (*p* = 0.003) in the multivariate analysis (Table 4). Forty-two (70%) of the 60 HGD/IMC samples with abnormal DNA content were associated with the development of persistent/recurrent HGD/IMC (*n* = 41) or EAC (*n* = 1), whereas only 6 (27%, all HGD/IMC) of the 22 remaining samples in the setting of normal DNA content were correlated with the detection of persistent/recurrent HGD/IMC within a mean follow-up time of 16 months (range: 1 month to 9.4 years) (Table 2). Similarly, Timmer et al. demonstrated that in HGD/IMC patients treated with ablation with or without preceding EMR, gains in multiple genomic loci as detected by FISH in endoscopic cytology brushings were associated with a lower probability of achieving complete eradication of HGD/IMC (HR = 0.57, *p* = 0.002) in a univariate analysis [34]. Multiple gains were observed in 32 (63%) of 51 patients who failed to achieve complete eradication, whereas they were found in 48 (37%) of 130 patients in whom complete eradication was achieved (*p* = 0.003). Krishnadath et al. also reported that all three patients (two with HGD and one with LGD), who were initially downstaged to LGD or non-dysplastic BE after PDT, eventually developed HGD and showed aneuploidy [35]. Before PDT, aneuploidy was found in two patients, one with HGD and the other with LGD. Taken together, these findings suggest that DNA content abnormality as detected by DNA flow cytometry (or alternative methods) may serve as a biomarker in identifying HGD/IMC patients who are at highest risk for persistent/recurrent HGD/IMC or EAC after each endoscopic session, potentially helping to optimize their treatment and follow-up strategies.

Reasons for decreased response to each endoscopic session in HGD/IMC patients with abnormal DNA content are unclear, but our results raise the possibility that the extent or apparent completeness (i.e., negative EMR margin) of endoscopic treatment may not always be sufficient to remove all high-risk neoplastic cells (i.e., those with abnormal DNA content). Indeed, Leedham et al. demonstrated that deep esophageal glands and their associated ducts that are capable of harboring *CDKN2A* and *TP53* mutations [36] can be found outside the depth of RFA (~0.5 mm) [37], which may permit regrowth of neoplasia prior to the patient’s next endoscopy. Also, Dvorak et al. reported that incomplete ablation was associated with increased p53 expression and Ki-67 proliferation index in non-dysplastic BE, whereas pre-ablation biopsies showed normal staining patterns [38]. It is certainly possible that neoplastic cells with DNA content abnormality and/or other genetic alterations may have a proliferation and survival advantage that allows them to be more resistant to endoscopic therapy, possibly contributing to increased Barrett’s epithelial thickness. This would provide additional support for the existing hypothesis that genetic and chromosomal abnormalities may be associated with persistent/recurrent neoplasia following endoscopic therapy [21, 22].

Our data confirm the previous observation that BE patients often require multiple endoscopic sessions to completely eradicate neoplasia [9, 13, 14]. In our cohort, the mean number of endoscopic sessions per patient was 3.4 (range: 1–18). More interestingly, based on the KM curves, 3-month, 6-month, 1-year, 3-year, and 6-year adjusted probabilities of persistent/recurrent HGD/IMC or EAC following each endoscopic session in the setting of abnormal DNA content were 31%, 56%, 67%, 79%, and 83%, respectively, whereas the corresponding probabilities in the setting of normal DNA content were 10%, 21%, 28%, 38%, and 43%, respectively (Fig. 3B; Table 3). This suggests that HGD/IMC patients, in the setting of normal DNA content, are more likely to require fewer endoscopic sessions to achieve complete eradication. Indeed, Wani et al. reported that baseline HGD/IMC and the number of endoscopic sessions required to achieve complete eradication of neoplasia and BE were significant predictors of any recurrence with odds ratios of 4.2 and 1.8, respectively [39]. The odds of BE and neoplasia recurrence increased by 78% with each additional endoscopic session. As such, if there is no or suboptimal response after a few endoscopic sessions (i.e., persistent/recurrent HGD/IMC despite 6–12 months of endoscopic therapy), alternative therapeutic strategies (i.e., higher dose RFA, different ablation technique, combined endoscopic modalities, or endoscopic submucosal dissection) [37, 40] as well as long-term follow-up with shorter surveillance intervals may be warranted. In support of this, we note that treatment with EMR alone was associated with an increased risk for persistent/recurrent HGD/IMC or EAC (HR = 2.7, *p* = 0.047) compared with other endoscopic modalities with or without preceding EMR in the multivariate analysis (Table 4).

Currently, surveillance practices after endoscopic therapy are informed by expert opinion alone [41]. Patients with baseline HGD/IMC are recommended to undergo frequent endoscopic follow-up: every 3 months for the first year, every 6 months in the second year, and annually thereafter. However, some authors have argued that the frequency of surveillance endoscopy should be lessened. For instance, using data from the United States Radiofrequency Ablation Registry (US RFA, 2004–2013) and the United Kingdom National Halo Registry, Cotton et al. recently developed three categories of risk based on baseline histologic grade prior to complete eradication (non-dysplastic BE/IND, LGD, and HGD/IMC) and modeled intervals to yield 0.1% risk of recurrence with EAC [42]. Using these risk prediction models, they proposed that patients with baseline HGD/IMC undergo surveillance at 3 months, 6 months, and 1 year after complete eradication, and annually thereafter. In this regard, we note that HGD/IMC patients with normal DNA content had lower rates of persistent/recurrent HGD/IMC. Therefore, lengthening follow-up intervals may be appropriate in these patients. In contrast, HGD/IMC patients with abnormal DNA content may warrant shorter surveillance intervals with long-term follow-up.

As for LGD, there has been a shift toward favoring endoscopic therapy in recent years, even though continued surveillance every 12 months is an acceptable approach [9–11, 41, 43, 44]. After complete eradication, surveillance endoscopy is recommended at 1 and 3 years for BE patients with a baseline diagnosis of LGD [9]. In this regard, we previously reported a significant correlation between abnormal DNA content and LGD patients who were subsequently found to have HGD or EAC within a year, with the estimated univariate and multivariate HRs of 7 and 18, respectively, from the Cox model [26]. This is consistent with our current finding that for the two LGD samples with normal DNA content, there was no evidence of neoplasia after RFA within a mean follow-up time of 27 months (range: 25–29 months) (Table 2). Normal flow cytometric results at baseline LGD could potentially enable clinicians to recommend surveillance rather than endoscopic therapy, whereas endoscopic therapy may be more appropriate in the setting of abnormal DNA content.

Consistent diagnoses and grading of dysplasia can be challenging, especially in the setting of intense acute/chronic inflammation. This is exemplified by a relatively high degree of interobserver variability among pathologists [27, 28, 45–47]. DNA flow cytometry can be helpful since features possibly altering the histologic interpretation (i.e., mucosal erosion, ulceration, and/or increased acute/chronic inflammation) do not cause aneuploidy or elevated 4N fraction [31, 48]. While over-diagnosis of HGD is not uncommon (up to 40% in one series [47]), abnormal flow cytometric results can serve as an objective marker to confirm a diagnosis of HGD/IMC with an estimated sensitivity of 77%. This close concordance of HGD/IMC with DNA content abnormality is consistent with our previous finding that up to 95% of HGD samples can show abnormal DNA content (versus 0% of non-dysplastic BE and 21% of LGD) [26]. In the previous study, 53% of HGD had concurrent or subsequent EAC detected on definite treatment for HGD (EMR or esophagectomy) or evidence of metastatic disease, which may explain the higher rate of DNA content abnormality when compared with the current study. Also, we note that two IND samples in the current cohort showed normal DNA content with no evidence of neoplasia within a mean follow-up time of 80 months (range: 46–115), suggesting that they most likely represented reactive changes rather than true dysplasia (Table 2). Furthermore, the lack of DNA content abnormality in eight control, non-dysplastic BE samples is in agreement with several previous studies also reporting a rate of 0% [26, 49, 50].

Our study has some potential limitations. First, although DNA content abnormality may be a marker of decreased response to each endoscopic session, its clinical implementation may require independent validation in a prospective setting. Second, we defined reduced response to each endoscopic session as the presence of neoplasia at the first or subsequent follow-up evaluation. One may argue that the high rate of persistent/recurrent HGD/IMC following each endoscopic session, especially during the first year, is likely due to incomplete endoscopic therapy, since multiple endoscopic sessions are usually needed before achieving complete eradication of neoplasia. However, our results suggest that DNA content abnormality could potentially serve as a biomarker to estimate the likelihood of persistence/recurrence after any single endoscopic session. Lastly, all the patients in our cohort were referred to or seen at UCSF Medical Center and San Francisco VA Health Care System which implies that referral bias cannot be excluded, but the direction of this potential bias is difficult to predict in this setting.

In conclusion, in BE patients with baseline HGD/IMC, both abnormal DNA content and treatment with EMR alone were significantly associated with persistent/recurrent HGD/IMC or EAC following each endoscopic session. The assessment of DNA content abnormality by DNA flow cytometry (or alternative methods) could be useful as an adjunct to standard histology in determining the effectiveness of each endoscopic session and, thus, optimizing both treatment and surveillance strategies for each patient. DNA content abnormality can also serve as a diagnostic marker of HGD/IMC with an estimated sensitivity of 77%. Identification of DNA content abnormality in the setting of HGD/IMC may warrant alternative therapeutic strategies as well as long-term follow-up with shorter surveillance intervals.

## Data Availability

All data generated or analyzed during this study are included in this published article.
